# Endothelial-monocyte activating polypeptide II disrupts alveolar epithelial type II to type I cell transdifferentiation

**DOI:** 10.1186/1465-9921-13-1

**Published:** 2012-01-03

**Authors:** Yao Chen, Susan K Legan, Anne Mahan, Janet Thornton, Haiming Xu, Margaret A Schwarz

**Affiliations:** 1 Department of Pediatrics, University of Texas Southwestern Medical Center at Dallas, Dallas, TX, USA

**Keywords:** EMAP II, alveolar epithelial cell, transdifferentiation

## Abstract

**Background:**

Distal alveolar morphogenesis is marked by differentiation of alveolar type (AT)-II to AT-I cells that give rise to the primary site of gas exchange, the alveolar/vascular interface. Endothelial-Monocyte Activating Polypeptide (EMAP) II, an endogenous protein with anti-angiogenic properties, profoundly disrupts distal lung neovascularization and alveolar formation during lung morphogenesis, and is robustly expressed in the dysplastic alveolar regions of infants with Bronchopulmonary dysplasia. Determination as to whether EMAP II has a direct or indirect affect on ATII→ATI trans-differentiation has not been explored.

**Method:**

In a controlled nonvascular environment, an *in vitro *model of ATII→ATI cell trans-differentiation was utilized to demonstrate the contribution that one vascular mediator has on distal epithelial cell differentiation.

**Results:**

Here, we show that EMAP II significantly blocked ATII→ATI cell transdifferentiation by increasing cellular apoptosis and inhibiting expression of ATI markers. Moreover, EMAP II-treated ATII cells displayed myofibroblast characteristics, including elevated cellular proliferation, increased actin cytoskeleton stress fibers and Rho-GTPase activity, and increased nuclear:cytoplasmic volume. However, EMAP II-treated cells did not express the myofibroblast markers desmin or αSMA.

**Conclusion:**

Our findings demonstrate that EMAP II interferes with ATII → ATI transdifferentiation resulting in a proliferating non-myofibroblast cell. These data identify the transdifferentiating alveolar cell as a possible target for EMAP II's induction of alveolar dysplasia.

## Introduction

Alveolar epithelial cells (AECs), located deep within the lung, have a pivotal role in gas exchange by acting in conjunction with the capillary bed to disperse oxygen throughout the body. Disruption of the distal alveolar lining of the lung through environmental or inflammatory induced injury results in the destruction of functional gas-exchanging alveolar type I (ATI) cells. Independent of the initial etiology, pathologic progression of acute lung injury (ALI) is the same marked by regions of scarring intermixed with alveolar damage, dysfunctional vasculature, and fibro-proliferative lung disease [1,2]. Within this process and essential to regeneration of gas-exchanging epithelial cells to satisfy the body's oxygen demands, is the regrowth of AECs. Recent studies suggest a paradigm shift in our understanding of distal lung repair. Although previously ATII cells were identified as an endogenous progenitor cell that gives rise only to gas-exchanging ATI cells, the ability of the ATII cell to function in a pluripotent manner was recently recognized. In response to local factors such as TGF-β expression, ATII cells can undergo an epithelial to mesenchymal transdifferentiation (EMT) to become myofibroblast [3,4]. Therefore, repopulation of the distal alveoli with gas-exchanging ATI cells following ALI is dependent on local growth factors that have the capability of redirecting differentiating ATII cells to myofibroblast thus contributing to the pathologic fibro-proliferative lung disease.

Our studies focus on one such vascular growth factor, Endothelial Monocyte Activating Polypeptide II (EMAP II). Although EMAP II's impact on the pathologic progression of hypoplastic lung disease has been well documented, little is known regarding the mechanisms that contribute to formation of the functional gas-exchanging ATI cells [5,6]. EMAP II, located on the cell surface, undergoes proteolytic cleavage to a mature ≈22-kDa form (mEMAP II) [7-9] that functions as a potent anti-angiogenic peptide [10,11]. Prevalent in early lung development, its expression is inversely correlated to periods of vascularization [12,13]. However excess amounts of mEMAP II delivered in a recombinant form to a murine allograft model of lung development profoundly disrupts not only vascular formation, but strikingly inhibits alveolar growth with a concomitant induction of distal alveolar apoptosis [5]. Furthermore, EMAP II expression is markedly increased in pathologic states associated with lung dysplasia such as in the distal alveoli of infants with Bronchopulmonary dysplasia (BPD) [6], LPS-induced acute lung injury [14], and emphysema [15]. Due to EMAP II's ability to inhibit distal alveoli formation and its elevation in disease processes where ATI cells are compromised, our studies focused on one of the properties associated with the regeneration of gas-exchanging ATI cells, ATII → ATI transdifferentiation. We demonstrate that EMAP II inhibits ATII → ATI differentiation. Furthermore, while EMAP II increased ATII cell apoptosis, there was also a concomitant increase in cellular proliferation. Associated with the increase in proliferation, F-actin bundles and Rho-GTPase activity were markedly increased. However, contrary to previous reports where F-actin and elevated Rho-GTPase activity is associated with EMT, EMAP II treated cells did not express the myofibroblast markers of desmin or αSMA. These studies indicate that EMAP II directly interferes with ATII → ATI transdifferentiation resulting in a non-myofibroblast undifferentiated cell, thus identifying the transdifferentiating cell as a possible target for EMAP II's induction of alveolar dysplasia.

## Materials and methods

### Cells

#### Primary alveolar epithelial cell isolation and AEC monolayer

AT II cells were isolated from adult Sprague-Dawley male rats (120-160 g) as previously described [16,17]. In brief, lungs were disassociated with elastase (1.5-2.0 U/ml, Worthington Biochemical, Freehold, NJ) and isolated based on their differential adhesion properties to IgG (Sigma, St. Louis, MO). Freshly isolated AT II cells were plated in a minimal defined serum-free medium (MDSF), (DMEM/F12 1:1, Sigma Aldrich, St. Louis, MO) on 4.67 cm2 tissue culture-treated polycarbonate filter cups (Transwell, Corning Incorporated, MA) in a humidified 5%CO2 incubator at 37°C. Cytospins of fresh cell isolates indicated 85-90% ATII cell purity determined by immunofluorescent staining for P180. Fibroblasts were selectively removed from cultures with the addition of a 100 μg/ml cis-OH-proline (Sigma Aldrich, St. Louis, MO) supplement for the first 48 hours [18]. Some cultures were treated daily with either vehicle (control, PBS) or EMAP II (3 mcg/ml) beginning on day 0 through day 8, while some cultures were treated for only the first 4 days of 8 treatment days. Animals were treated in accordance with the guidelines of and with the approval of the University of Texas, Southwestern Medical Center Institutional Animal Care and Use Committee.

### Protein analysis

#### Western analysis

For Western analysis, cells were lysed in 50 mM Tris pH 7.4, 0.9 N NaCl, 1% NP-40, and 0.01% NaN3, in the presence of the protease inhibitors (aprotinin 20 μg/ml, leupeptin 20 μg/ml, and pepstatin A 20 μg/ml), and stored at -70°C. Homogenates were cleared by centrifugation at 14,000 × g for 20 minutes, the protein concentration determined by Bradford analysis (BioRad Hercules, CA), and the samples normalized by protein content. Equal amounts of protein were electrophoresed on a 10% SDS-PAGE gel, transferred to nitrocellulose membranes, blocked overnight in a casein-based blocking solution (Boehringer-Mannheim, Indianapolis, IN), and probed with primary antibody followed by horseradish-peroxidase-conjugated anti-mouse (Jackson ImmunoResearch Laboratories, West Grove, PA) and anti-rabbit IgG antibodies were purchased from Thermo Scientific, Rockford, IL. Specific binding was detected using a chemiluminescence substrate (Pierce, Rockford, IL) and XAR-5 film (Eastman Kodak, Rochester, NY). Quantitative analysis was accomplished using Quantity One Software and samples were normalized against actin or tubulin.

#### Rho Kinase Assay

Active Rho Kinase was assessed using an EZ-Detect™ Rho Activation Kit (Pierce, Rockford, IL) based on Rhotekin Rho-binding domain. Protein was isolated according to manufacture protocol, protein concentration assessed, and Rho Kinase activity examined on equal protein amounts using Western Blot analysis. GTP, GDP, and unfractionated cell lysate were used as controls. Semi-quantitative densitometry was carried out as previously described [19].

### Histological analysis

#### Immunofluorescence

Alveolar epithelial cell monolayers were rinsed with ice-cold phosphate-buffered saline (PBS) twice and fixed in 4% paraformalderhyde overnight at 4 degree. Cells were permeabilized in 0.1%Triton X-100, followed by blocking with CAS (Invitrogen, Carlsbad, CA) and exposed to the primary antibody 1 hr [anti-β-catenin (Santa Cruz Biotechnology,), anti-Ki67 (Abcam, Cambridge, MA), anti-ZO-1(Invitrogen, Carlsbad, CA), anti-Lamellar body P180 (Covance, Berkeley, CA), anti-AQP5 (Chemicon, Temecula, CA), anti-fibronectin (Santa Cruz Biotechnology, Santa Cruz, CA), anti-actin or anti-F-actin (Invitrogen,)] as per manufactures instructions. Following washing with PBS, tissues were exposed to the appropriate secondary Cy3 or AlexaFluor 488 fluorescent antibody (Chemicon, Temecula, CA and Molecular Probes Invitrogen, Carlsbad, CA) for 1 hour. This was followed by a six minute incubation with membrane permeable DAPI (4',6-Diamidino-2-phenyindole 5 mg/ml at 1:1,000 dilution, Invitrogen), rinsing with PBS and mounting. TUNEL staining was performed using an ApoAlert DNA Fragmentation Assay kit (Clontech, Mountain View, CA) according to the manufacturer's instructions. Positive TUNEL staining (green staining) cells were characterized as apoptotic cells and 10 randomly selected microscopic fields in each group were used to calculate the percentage of TUNEL positive cells. Similarly positive Ki67 cells were examined in 10 randomly selected fields. Importantly total cell number was calculated using DAPI (nuclear) counting in the same field and the positive TUNEL or Ki67 cells were reported as percent of cells present. The signal was viewed by fluorescent microscopy at the appropriate wavelength for the secondary antibody on an IX81 Olympus microscope and images captured with a Hamamatsu Orca digital camera with a DSU spinning confocal unit using Slidebook software.

### Statistical analysis

Statistical analysis was performed using a Student's T test or ANOVA on the software PRISM 4.0 for MacIntosh (GraphPad Software, Inc., San Diego, CA).

## Results

### EMAP II treatment mediates ATII → ATI trans-differentiation

**Figure 1:**
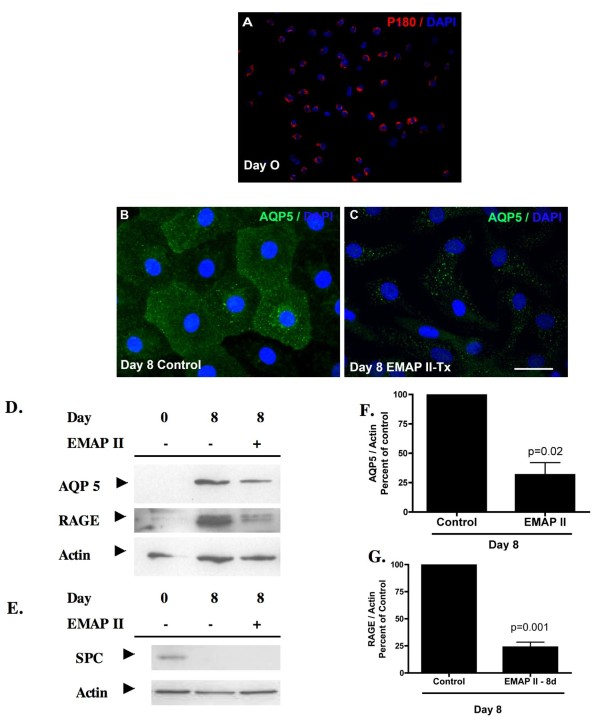
**EMAP II inhibits AT II **→ **AT1 transdifferentiation**. Aquaporin 5 (AQP5) expression was examined 8 days following isolation of AT II cells using IgG panning (A - p180 lamellar body positive, cy3; G - surfactant protein C positive, day 0). Immunofluorescence and Western blot analysis for AQP5 indicated that AT II cells transdifferentiate into AT1 cells over an eight-day period (B - FITC, D, E). AT II cells subjected to EMAP II for 8 days in culture have a marked reduction in AQP5 and RAGE expression (C, D, F, G) as compared to control (B, D, F, G). Neither cell population had surfactant protein C (SPC) expression after 8 days in culture (E). DAPI denotes nuclear staining. Scale bar = A - 37.5 μ, B-D-25 μ.

Although angiogenesis is widely described as influencing distal pulmonary morphogenesis, the molecular events during vessel formation that drive pulmonary development have not been determined. Previous studies have shown that the anti-angiogenic protein EMAP II profoundly disrupts alveolar-capillary growth and is highly expressed in distal alveoli of hypoplastic lungs [5,6]. In a controlled, nonvascular environment, we utilized an *in vitro *model of ATII → ATI cell trans-differentiation [16,17,20] to demonstrate the contribution that one vascular mediator has on distal epithelial cell differentiation. Freshly isolated ATII cells from dissociated rat lungs were subjected to IgG panning and determined to be 85-90% pure by lamellar body expression (P180, Figure 1A), were plated on polycarbonate filters in serum-free medium. Cells were exposed to either vehicle or the anti-angiogenic protein EMAP II on days 0-8 and examined for ATI markers. As can be seen in Figure 1B, ATII cells gradually flattened, acquired an epithelial like polygonal shape, and express the ATI water channel membrane protein Aquaporin 5 (AQP5) on day eight. In contrast, ATII cells treated with EMAP II exhibited a marked reduction in AQP5 protein as determined by immunofluorescence and confirmed by Western Analysis (Figure 1C, D, F, G, Student's T-test p = 0.02) (n = 5 on separate occasions) as compared to control (Figure 1B, D, F, G). RAGE (Receptor for Advanced Glycation Endproducts), a second AT1 marker was also significantly reduced (D, G, n = 5 on separate occasions). Importantly, no surfactant protein C (SPC) expression was noted in cells at day 8 in either the control or EMAP II cells (Figure 1E) suggesting that in all conditions at day 8 the cells no longer express the ATII protein SPC. Studies over 8 days where cells were treated with EMAP II for only the first 4 days and then EMAP II was replaced with vehicle for the last 4 days prior to harvest, these cells demonstrated a 40% reduction in AQP5 and RAGE protein expression (data not shown). These data suggest that EMAP II inhibits ATII→ATI trans-differentiation. We next explored alternative pathways of ATII trans-differentiation.

### EMAP II induces apoptosis in trans-differentiating alveolar cells

**Figure 2:**
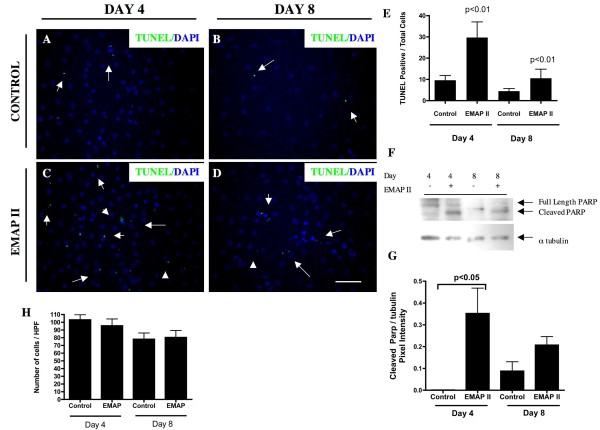
**Apoptosis is increased by EMAP II in transdifferentiating cells**. Isolated, AT II cells were examined at days 4 and 8 for apoptosis using an immunofluorescent TUNEL assay. Cells treated with EMAP II had a marked increase in apoptotic cells at days 4 and 8 (C, D, and E) as compared to control (A, B, and E). Western blot analysis of the caspase cleaved PARP-1 indicated a marked increase in PARP-1 cleavage in EMAP II treated cells as compared to control (F, G). DAPI denotes nuclear staining. Despite an increase in apoptosis, total cell number remained unchanged between control and EMAP II treated cells (H). Scale bar = 37.5 μ.

Previous studies have shown that EMAP II inhibits distal ATII and ATI cells during lung morphogenesis [5]. In addition to distal lung lacking ATII and ATI markers, EMAP II induced apoptosis in the distal alveoli of the developing lung and *in vitro *lung co-culture epithelial cysts [5,21]. One response of alveolar epithelial cells to injury is apoptosis [3]. We performed TUNEL analysis on trans-differentiating ATII → ATI cells to determine if EMAP II had the ability to induce apoptosis in differentiating alveolar cells. Consistent with previous *in vivo *and *in vitro *lung co-culture [5,21], isolated trans-differentiating ATII cells had a marked increase in TUNEL positive cells in the presence of EMAP II treatment (Figure 2C-E, p < 0.01, Student's T-test) (n = 11 high powered field, performed on 3 different occasions) as compared to control (Figure 2A, B, E). Apoptosis was confirmed by PARP-1 cleavage (Figure 2F, G, p < 0.05, One-way Anova with Post Hoc Tukey's test). Notably, only portions of the total cell population were undergoing apoptosis. We next explored alternative ATII trans-differentiating pathways.

### Proliferation and nuclear volume are increased by EMAP II

**Figure 3:**
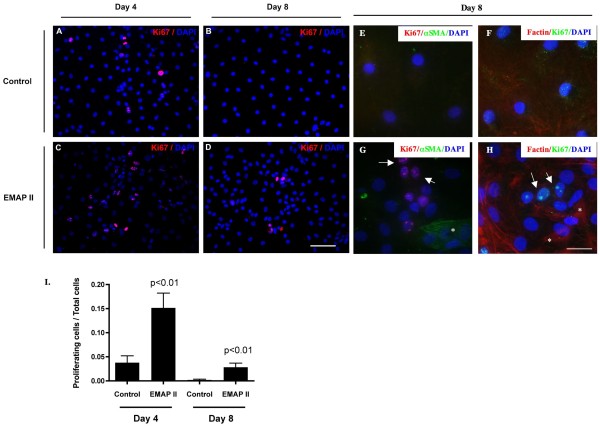
**EMAP II increases proliferation in non-myofibroblast cells**. Proliferation was assessed using immunofluorescence for Ki67 in isolated AT II cells in culture at days 4 and 8. EMAP II significantly increased proliferation at days 4 and 8 (C, D, I) as compared to control (A, B, I). Co-localization of Ki67 with αSMA (E-control, G-EMAP II treated) and F actin (F-control, H-EMAP II treated) indicates that the proliferating cell population was negative for myofibroblast markers. DAPI denotes nuclear staining. Scale bar = 37.5 μ (A-D), 25 μ (E-H).

**Figure 4:**
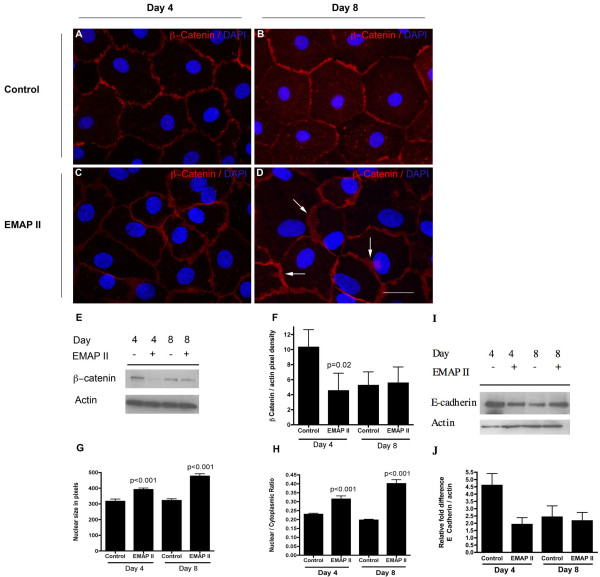
**Decreased Beta-catenin expression corresponds with marked membrane ruffling, increase in nuclear size and nuclear:cytoplasmic ratio in EMAP II treated cells**. Immunofluorescent staining of AT II cellular membranes indicate that transdifferentiating AT II cells develop a cobblestone appearance of epithelial like polygonal shaped cells (A, B). Transdifferentiating AT II cells treated with EMAP II lack the polygonal shape (C, D), exhibit membrane ruffling (arrows, D), and have decreased β-catenin expression of at day 4 (E, F). In addition, EMAP II treated cells demonstrated a larger nuclear size and nuclear:cytoplasmic ratio as compared to controls (G, H). DAPI denotes nuclear staining. E-cadherin expression was not significantly different between EMAP II and control cells (I, J) Scale bar = 25 μ.

In spite of the fact that there was a marked increase in apoptosis in the EMAP II treated cells, there was no difference in total cell counts between EMAP II and Control treated cells (Figure 2H). One way to maintain an equivalent cell number in the presence of on going cell death is cellular proliferation. Although trans-differentiating ATII → ATI cells are not known to proliferate [22], we examined whether the equivalent cell numbers between groups was due to cellular proliferation. Histological analysis of the cell proliferative marker Ki67 in 10 high powered fields on three different occasions indicates that trans-differentiating ATII cells treated with EMAP II have a significant increase in proliferation on days 4 and 8 in culture (Figure 3C, D, I, p < 0.01, Student's T-test, n = 10 high powered fields) as compared to control (Figure 3A, B, I). Importantly, EMAP II induced cellular proliferation was limited to non-myofibroblast type cells as the Ki67 cells did not co-express F-actin bundles nor α smooth muscle actin (Figure 3G, H, * - αSMA and F-actin bundles, arrow - Ki67 positive cells). In conjunction with the increase in proliferation, we examined the cellular distribution of a member of the adherens junction proteins, β-catenin which is responsible for stopping epithelial cell division through transmission of a contact inhibition signal [23]. EMAP II treated cells demonstrated a marked loss of the cobblestone appearance of epithelial like polygonal shaped cells (Figure 4C, D), a decrease in β-catenin expression at day 4 (Figure 4E, F) (p = 0.02, Student's T-test, n = 4 on 4 separate occasions), and an increase in β-catenin membrane ruffling (Figure 4C-D, arrows) as compared to control (Figure 4A, B, E, F) (p < 0.01, ANOVA, n = 3 on separate occasions). Zona occludens (data not shown) and E-cadherin expression (Figure 4I, J, One-way ANOVA with Tukey's post-hoc test) was similar between the two cell populations (n = 4 on 4 separate occasions). In addition to a loss of cobblestone polygonal shape, the cell nuclear volume and nuclear:cytoplasmic ratio were noted to be markedly increased in trans-differentiating AT II cells treated with EMAP II (Figure 4G, H) as compared to control (p < 0.01, One-way ANOVA with Tukey's post-hoc test, n = 7, representative of 4 separate occasions).

### F-actin bundles and Rho-Kinase activity are increased by EMAP II while αSMA and desmin activity are suppressed

**Figure 5:**
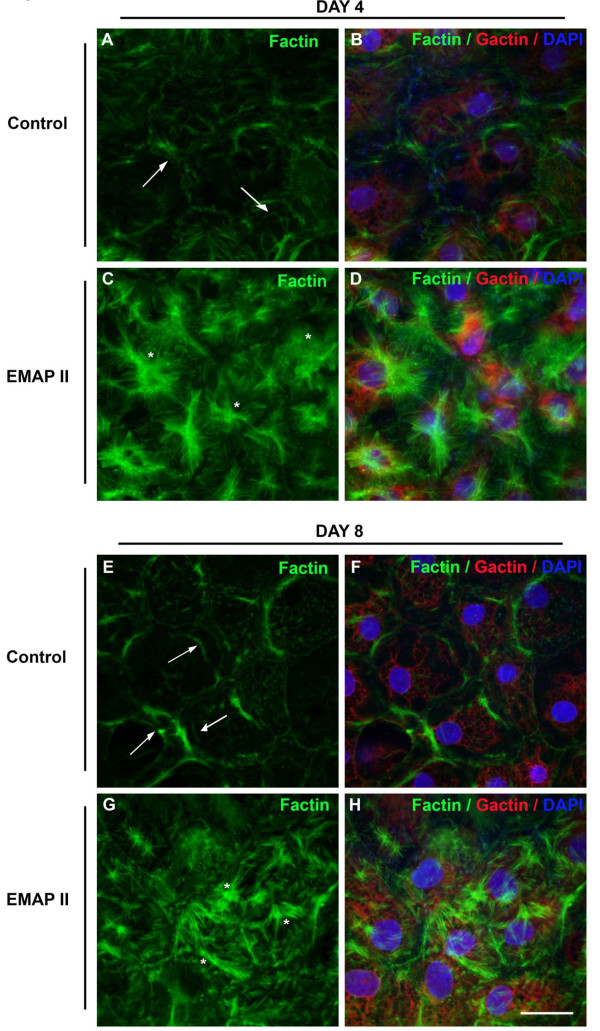
**EMAP II inhibited actin cortical bands and increased actin bundles in transdifferentiating AT II cells**. F-actin at days 4 and 8 is distributed in cortical bands in differentiating AT II cells (arrows, A, B and E, F, FITC). Transdifferentiating cells treated with EMAP II lack cortical bands and demonstrate marked increase in actin bundles (stars, C, D and G, H, FITC). G-actin is revealed with cy3 and DAPI denotes nuclear staining. Scale bar = 25 μ.

**Figure 6:**
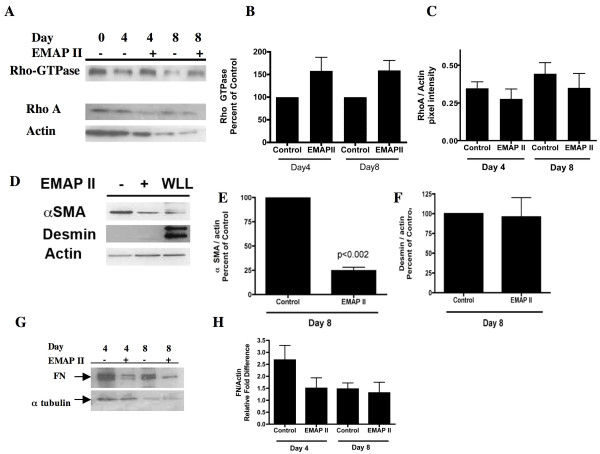
**EMAP II increases Rho-GTPase expression while αSMA and desmin expression are decreased in transdifferentiating AT II cells**. Rho-GTPase activity was assessed in AT II cells in culture at 0, 4, and 8 days using a Rhotekin RBD probe to isolate active GTP-Rho. EMAP II treatment increased Rho-GTPase activity as compared to controls (A, B). However total Rho A levels normalized to actin in ATII cells in cultures treated with EMAP II were unchanged as compared to control (A, C). After 8 days in culture, αSMA actin levels were markedly decreased as compared to control (D, E) and desmin was minimally expressed in EMAP II treated cells (D, F). Protein expression was normalized to actin loading controls and whole lung lysate (WLL) was used as an addition loading control (D). Fibronectin expression (FN) was reduced following EMAP II treatment (G, H, normalized to alpha-tubulin loading control).

As epithelial-mesenchymal cell transition (EMT) is associated with active membrane ruffling of bound β-catenin [24,25], increases in nuclear:cytoplasmic volume [26], and loss of the cobblestone appearance of polygonal shaped cells [27], we examined EMAP II treated cells for signs of EMT. Actin cytoskeleton in control cells demonstrated cortical bands at days 4 and 8 (Figure 5A, B, Figure 5E, F, arrows). In contrast, the F actin in EMAP II treated cells displayed actin bundles at all time points consistent with the cytoskeletal structure found in myofibroblast (Figure 5C, D, G, H, stars). As F-actin deposition and cytoskeletal rearrangement is a downstream marker of Rho-GTPase activity, Rho-GTPase activity was examined. Further supporting EMT of cells into a myofibroblast phenotype, EMAP II treatment markedly increased Rho-GTPase activity as compared to control (Figure 6A, B, One-way ANOVA with Tukey's post-hoc test, 6A, C- Total RhoA expression was not significantly different between groups, One-way ANOVA with Tukey's post-hoc test). However, Western blot analysis indicates that EMAP II suppresses expression of the myofibroblast markers α smooth muscle actin (SMA) (Figure 6D, E) (p < 0.002, Student's T-test) and desmin (Figure 6D, F, Student's T-test) (n = 3 on different occasions, day 4 analysis showed no expression of αSMA or desmin in either treatment group). In addition, the extracellular matrix protein FN was decreased in cells treated with EMAP II (Figure 6G, H, NS, n = 4 on different occasions, One-way ANOVA with Tukey's post-hoc test). Taken together these data suggests that EMAP II inhibits differentiating AT II cells from becoming AT1 cells or myofibroblast resulting in a cell either undergoing apoptosis or assuming an undifferentiated cell phenotype.

## Discussion

The mature form of EMAP II (mEMAP II) has been shown to induce distal alveolar dysplasia, however the mechanism associated with its inhibition of alveoli is poorly understood. What is clear is that in addition to a marked reduction in vessel formation, there is a concomitant reduction in alveolar cells. This raised the possibility that in addition to EMAP II's inhibition of vasculature having an indirect suppressor effect on alveolar cells, that EMAP II might indeed directly inhibit ATI cell formation. Examination of the cell population thought to give rise to ATI cells, ATII cells, shows that mEMAP II blocks transdifferentiation of the ATII cells into ATI cells in an avascular model. Furthermore, we determined that ATII cells exposed to EMAP II either underwent apoptosis or expressed several phenotypic markers of EMT. However, in contrast to previous studies where cells express markers of EMT, EMAP II treated ATII cells did not express the myofibroblast markers αSMA or desmin. These findings indicate that in a non-vascular system, EMAP II alters ATII → ATI transdifferentiation resulting in the formation of a non-myofibroblast undifferentiated cell population. These findings support a role for mEMAP II that reaches beyond the endothelium in distal alveolar differentiation.

Cell fate is influenced by growth factors and determines the regeneration of functional air-exchanging alveolar cells or myofibroblastic cells following lung injury. Previous observations showed that ATII transdifferentiation does occur *in vitro *and leads to one of three distinct pathways which results in ATI, myofibroblast, or apoptotic cell populations [3,4]. A cellular model affords a unique opportunity to explore the mechanisms influencing cellular fate. Previous studies suggest that the naturally occurring protein EMAP II may have a role in distal lung repair as EMAP II is highly expressed in the distal lung in patients with BPD [6], emphysema [15], and following LPS induced lung injury [14]. In these circumstances of disease and lung damage, such as that seen in smoke induced lung damage, EMAP II plays a significant and leading role [15]. Further determination on how EMAP II mechanistically influences distal alveolar differentiation versus death are essential in being able to develop therapeutic strategies to combat lung damage. However, it is not known whether the elevated levels of EMAP II influence distal alveolar regeneration processes. Importantly, in line with those seen when other angiogenic mediators [28-32], exogenous mEMAP II inhibited vascular and distal alveolar cell formation without clarifying whether its affect was through a direct influence on alveolar cells or an indirect consequence of the lack of vasculature. While EMAP II is not known to be involved in ATII→ATI transdifferentiation, in these avascular studies we show that mEMAP II directly mediates *in vitro *ATII cell differentiation. Specifically in conjunction with a loss of ATII and ATI cell markers, there was an increase in apoptosis, consistent with our distal alveolar findings using a fetal lung explant model [5]. Interestingly, EMAP II induced apoptosis in a limited cell population. This is similar to our previous findings in endothelial cells where EMAP II induced apoptosis only in the actively dividing endothelial cell population [10]. As fibronectin levels were decreased in the differentiating ATII cells and fibronectin has been shown to enhance pulmonary ATII epithelial cytoskeleton and tight junction organization [33], we speculate that the decrease in fibronectin results in a reduction in tight junction organization making some cells vulnerable to apoptosis. These mechanistic questions are the focus of our ongoing studies. Taken together, these findings suggest that EMAP II has a greater role in lung that extends beyond the endothelium.

In contrast to the influence of other growth factors such as TGFβ induced ATII→ myofibroblast transdifferentiation [3,4], EMAP II-treatment results in the emergence of an as to yet unidentified cell population. We thus explored a potential alternative transdifferentiation pathway underlying EMAP II associated loss of ATI cell markers. While this cell population has some of the hallmark characteristics of a cell undergoing TGFβ induced EMT, increased nuclear/cytoplasmic volume, changes in nuclear shape [34], loss of cobblestone appearance [27], active ruffling of β-catenin, elevation of rho kinase activity [25,35] and actin cytoskeleton dynamics consistent with myofibroblast morphology, the resulting cell population lacks expression of the classic myofibroblast biomarkers αSMA and desmin [36,37]. Interestingly, despite the structural similarities between EMAP II treated cells and those undergoing EMT, TGFβ expression was not increased by EMAP II (data not shown). These findings suggest that EMAP II induces the emergence of a cell population whose role is yet to identified. While not previously identified in alveolar epithelial cell transdifferentiation, there is precedence for intermediate cell populations that undergo 'partial EMT' to exist during epithelial trans differentiation [38]. The role of EMAP II in epithelial transdifferentiation is the focus of our ongoing studies.

Proteolytic cleavage from the cell surface to a ≈ 22-kDa form (mEMAP II) [39], endogenous EMAP II functions as a potent anti-angiogenic peptide in both normal physiologic lung development [5,12,21] and pathologic tumor regulation [10,11,40-42]. A fundamental role in lung development is supported by EMAP II expression being inversely correlated to periods of vascularization [12,13] and presentation of recombinant EMAP II in a murine allograft model of lung development profoundly disrupts alveolar-capillary growth [5]. Furthermore the impact of EMAP II on the pathologic progression of lung disease has been well established while the mechanisms of how EMAP II facilitates distal lung dysplasia are poorly understood. Mechanistically, EMAP II functions by inhibiting binding of the α5β1 integrin to its extracellular ligand fibronectin. This inhibition results in delayed cell spreading and the disassembly of the cytoskeleton architecture of actin fiber networks, and fibronectin matrix [42,43]. Previous studies in Xenopus models support a role for α5β1 integrin regulation of spreading in non-pulmonary epithelial transdifferentiation. During Xenopus gastrulation, epithelial cell spreading is dependent on the cellular engagement of the fibronectin fibrils via the cells α5β1 integrin receptor and this initiates spatial cues that assign cellular polarity and differentiation [44,45]. Once fibronectin is engaged to the α5β1 receptor, this facilitates a cascade of events resulting in the formation of the complex 3-D mesh like fibrils in the extracellular space known as fibrillogenesis. In ATII cells a role for fibronectin has been identified. Immortalized pulmonary epithelial cells demonstrate adhesion affinity to fibronectin (rat type II cell lines - LM5 and alveolar cell carcinoma cells - A549) [46] while fibronectin enhances pulmonary ATII epithelial cytoskeleton actin and tight junction organization [33]. Analysis of EMAP II's ability to alter actin cytoskeletal organization suggests that there is a marked increase in actin bundles despite a significant decline in total cell fibronectin (Figure 6) and no change in α5β1 integrin expression. This finding is inconsistent with our previous observations in endothelial cells where EMAP II inhibits actin cytoskeleton formation [43]. What is not clear is whether epithelial transdifferentiation is dependent on specific extracellular matrix components and whether or not EMAP II alters these factors. This area of investigation is part of our ongoing studies.

## Conclusion

In conclusion, our studies indicate that the angiogenic mediator EMAP II influences ATII→ATI trans-differentiation. Understanding the role that this EMAP II has in the regulation of distal alveolar development and alveolar cell regeneration can allow further insight into the repair process that gives rise to an essential and functional gas-exchanging alveolar type I.

## Abbreviations

EMAP II: Endothelial Monocyte Activating Polypeptide II; AECs: alveolar epithelial cells; ATII: alveolar type II cell; ATI: alveolar type I cell; EMT: epithelial mesenchymal transition
